# Ultrasound evaluation of the morphometric patterns of lymph nodes of the head and neck in young and middle-aged individuals*

**DOI:** 10.1590/0100-3984.2015.0002

**Published:** 2016

**Authors:** Beatriz Ogassavara, Raul Renato Tucunduva Neto, Romeu Rodrigues de Souza, Maria José Tucunduva

**Affiliations:** 1 Intern at the Hospital do Servidor Público Estadual de São Paulo and at the Faculdade de Medicina da Universidade Cidade de São Paulo (Unicid), São Paulo, SP, Brazil.; 2 MD, Sonographer at the Clínica Tucunduva, Jundiaí, SP, Brazil.; 3 Tenured Professor of the Graduate School at the Universidade São Judas Tadeu (USJT), São Paulo, SP, Brazil.; 4 Tenured Professor in the Faculdade de Medicina da Universidade Cidade de São Paulo (Unicid), São Paulo, SP, Brazil.

**Keywords:** Lymph nodes/anatomy & histology, Radiology, Diagnostic imaging

## Abstract

**Objective:**

To show the morphometric patterns of lymph nodes of the head and neck, evaluating their number, shape, dimensions, hilum, and cortex, through the use of ultrasound examination of the neck.

**Materials and Methods:**

We analyzed 400 right and left lymph nodes in a group of 20 healthy young and middle-aged individuals of both genders.

**Results:**

In the ultrasound examination, we observed the following lymph nodes: mastoid; parotid (superficial, extraglandular, and intraglandular); submandibular (preglandular, retroglandular, and intracapsular); submental; and cervical (anterior and posterior). Although some individuals had up to seven lymph nodes in the same region, most had only two to three per region. The smallest lymph node diameter observed was 0.4 cm, and the largest was 2.7 cm. Most lymph nodes showed an elongated or oval shape. Most of the lymph node hila were echogenic, although a few were hyperechoic. However, the cortex was clearly hypoechoic in all of the lymph nodes evaluated.

**Conclusion:**

Ultrasound examination of healthy individuals allowed the characteristics of the lymph nodes of the head and neck to be observed clearly, which could provide a basis for the analysis of patients with diseases of these lymph nodes.

## Abstract (pt)

**Objetivo:**

Fornecer padrão morfoquantitativo ultrassonográfico dos linfonodos da cabeça e pescoço, mediante avaliação do seu número, forma, dimensões, hilo e córtex, como base para diagnóstico de alterações nestes parâmetros.

**Materiais e Métodos:**

Foram analisados 400 linfonodos dos lados direito e esquerdo de 20 indivíduos, homens e mulheres, jovens e de meia-idade.

**Resultados:**

Os linfonodos observados no exame ultrassonográfico foram: mastóideos, parotídeos superficiais, parotídeos extraglandulares, parotídeos intraglandulares, submandibulares pré-glandulares, retroglandulares e intracapsulares, submentuais, cervicais anteriores e posteriores. Quanto ao número de linfonodos, alguns indivíduos apresentaram até sete em uma mesma região, mas a maioria apresentou de dois a três linfonodos por região. O menor diâmetro dos linfonodos foi 0,4 cm e o maior foi 2,7 cm. A maioria dos linfonodos apresentou forma alongada ou ovalada. O hilo dos linfonodos apresentou-se geralmente ecogênico e poucas vezes hiperecogênico. De modo geral, o córtex dos linfonodos mostrou-se muito pouco ecogênico.

**Conclusão:**

A ultrassonografia de indivíduos sadios permitiu uma clara observação das características dos linfonodos da região da cabeça e pescoço, que podem ser importantes para análise de pacientes portadores de afecções desses linfonodos.

## INTRODUCTION

Knowledge of the location and characteristics of the lymph nodes of the head and neck is essential for the recognition of diseases such as tuberculosis, sarcoidosis, histoplasmosis, Hodgkin lymphoma, extrathyroid tumor metastases (to the lung, breast, esophagus, larynx, or other sites), and differentiated thyroid cancer^(1)^. The use of imaging methods to evaluate the characteristics of those lymph nodes assists the physician in the diagnostic process^(2)^, given that the clinical examination alone is often insufficient for a proper evaluation. The use of ultrasound allows aspects such as the number, shape, size, hilum, and cortex of the lymph nodes, as well as necrosis, calcification, extracapsular spread, and vascularization, to be evaluated^(3)^.

The lymph nodes of the head and neck can be superficial or can be located deep within the adjacent tissues. The superficial lymph nodes of the head are divided into five groups: occipital, mastoid, preauricular, superficial parotid, and facial. They are further subdivided into zygomatic, buccinator, nasolabial, and mandibular. The deep lymph nodes of the head, which are not palpable during extraoral examination, are classified as deep parotid or retropharyngeal^(4)^.

The superficial lymph nodes of the neck comprise the submental, superficial anterior cervical, superficial lateral cervical, and submandibular. The deep lymph nodes of the neck are distributed along the internal jugular vein, below the sternocleidomastoid muscle, ranging in number from 15 to 30. These nodes, which can be palpated, are divided into superior and inferior^(4)^.

On ultrasound, benign lymph nodes generally have an oval or elongated shape, with an echogenic hilum, and range from 0.1 to 2.5 cm in length, unless they contain calcifications or show cystic degeneration^(3,5)^. In contrast, those in which metastasis is strongly suspected are rounded, generally hypoechoic, with loss of hilum, and can present calcifications or cystic degeneration. Such lymph nodes are usually located in the lower third of the neck^(6)^.

The objective of this study was to evaluate the morphological and quantitative characteristics of the lymph nodes of the face and neck, in healthy subjects, by ultrasound.

## MATERIALS AND METHODS

A total of 20 volunteers underwent B-mode ultrasound examination. The study was approved by the Research Ethics Committee of the institution. All participants gave written informed consent.

The images were obtained with a Toshiba MX ultrasound system (Toshiba Medical Systems, Tokyo, Japan) with a 5-12 MHz linear transducer (12L5; Toshiba Medical Systems) and a convex transducer. A thin gel film was used between the transducer and the latex protector. The sample consisted of 20 apparently healthy subjects without symptoms of lymphadenopathy-10 men and 10 women, between 20 and 50 years of age (mean age, 24.2 years).

We measured the largest diameter (in cm) of lymph nodes on the right and left sides, examining a total of 400 lymph nodes: mastoid; parotid (superficial, extraglandular, and intraglandular); submandibular (preglandular, retroglandular, and intracapsular); submental; and cervical (anterior and posterior). Quantitative data were analyzed using the SPSS Statistics software package (IBM Corp., Armonk, NY, USA), and the level of significance was set at *p* < 0.05.

## RESULTS

**Figure 1:**
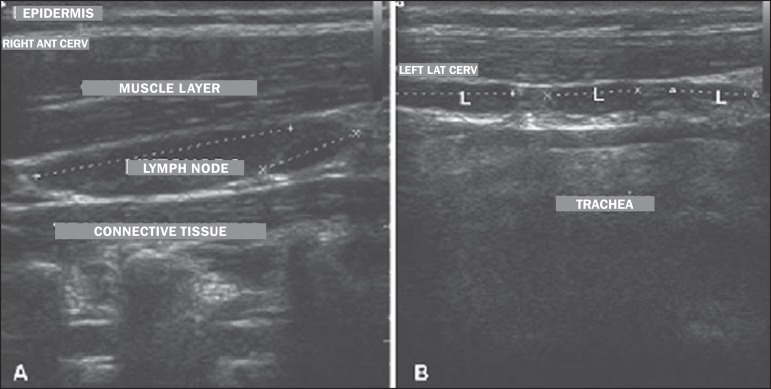
Ultrasound of female subjects (**A**) and male subjects (**B**) showing the location of the lymph nodes in relation to surrounding structures. The dotted line indicates the measurement of the largest diameter. L, lymph node.

Figure 1 shows ultrasound images of two of the subjects examined, showing the appearance of the lymph nodes evaluated. On ultrasound, the lymph nodes appeared elongated with an echogenic hilum and no abnormalities. The lymph nodes that were easily identified on ultrasound were the mastoid, parotid (extraglandular and intraglandular), submandibular (preglandular, retroglandular, and intracapsular), submental, and cervical (anterior and posterior) lymph nodes. The superficial parotid lymph node was observed in only one female subject.

Table 1 shows the mean values and other statistical parameters for the largest diameter of the lymph nodes in each region analyzed in both sexes.

**Table 1:** Measurements (largest diameters) of the lymph nodes on the right and left sides within the five regions studied, in male and female subjects.

	M			SP			EP			IP			PgS			RgS			IcS			SubMe			AC			PC	
Parameters	R	L		R	L		R	L		R	L		R	L		R	L		R	L		R	L		R	L		R	L
Overall mean	0.7	0.7		0.9	-		1.4	0.8		0.8	0.7		0.9	1.1		1.4	1.2		1.0	1.1		0.7	0.7		1.1	1.2		1.1	1.1
Median	0.8	0.6		0.9	-		1.1	0.8		0.7	0.7		0.9	1.0		1.3	1.3		1.0	1.0		0.7	0.7		1.0	1.2		1.0	1.0
Mode	0.8	0.6		-	-		-	-		0.6	0.5		0.7	0.9		2.0	1.7		0.9	1.0		0.7	0.7		1.2	1.0		1.2	1.0
Standard deviation	0.4	0.3		0.2	0.0		0.7	0.2		0.4	0.3		0.5	0.6		0.7	0.5		0.6	0.5		0.3	0.4		0.3	0.3		0.6	0.4
Variation	0.2	0.1		0.0	0.0		0.5	0.1		0.1	0.1		0.2	0.4		0.4	0.2		0.3	0.3		0.1	0.1		0.1	0.1		0.3	0.2
Females																													
Mean	0.7	0.6		0.9	-		1.7	-		0.7	0.6		0.8	0.9		1.5	1.2		1.1	1.2		0.6	0.7		1.1	1.3		1.0	1.1
Median	0.7	0.6		0.9	-		1.7	-		0.6	0.6		0.8	0.9		1.6	1.3		0.9	1.1		0.6	0.8		1.0	1.3		1.0	1.0
Mode	-	0.6		-	-		-	-		0.6	0.5		-	-		2.0	-		0.9	-		-	-		1.2	1.0		-	1.0
Standard deviation	0.4	0.3		0.3	0.0		0.9	0.0		0.3	0.2		0.3	0.4		0.8	0.5		0.6	0.6		0.3	0.4		0.3	0.3		0.5	0.4
Variation	0.1	0.1		0.1	0.0		0.7	0.0		0.1	0.0		0.1	0.1		0.6	0.2		0.4	0.4		0.1	0.2		0.1	0.1		0.2	0.2
Males																													
Mean	0.9	0.7		-	-		1.2	0.8		0.9	0.9		0.9	1.1		1.3	1.2		1.0	0.9		0.7	0.8		1.1	1.2		1.2	1.1
Median	0.9	0.7		-	-		1.1	0.8		0.8	0.9		1.0	1.1		1.3	1.3		1.0	0.9		0.7	0.7		1.1	1.1		1.0	1.1
Mode	-	-		-	-		-	-		0.6	1.0		1.0	1.2		1.3	-		1.0	-		0.7	-		-	-		-	-
Standard deviation	0.4	0.4		0.0	0.0		0.9	0.3		0.4	0.4		0.5	0.5		0.4	0.2		0.3	0.4		0.4	0.4		0.2	0.3		0.7	0.3
Variation	0.2	0.1		0.0	0.0		0.4	0.1		0.2	0.2		0.3	0.3		0.3	0.2		0.2	0.1		0.1	0.1		0.1	0.1		0.4	0.1

M, mastoid (lymph nodes); SP, superficial parotid (lymph nodes); EP, extraglandular parotid (lymph nodes); IP, intraglandular parotid (lymph nodes); PgS, preglandular submandibular (lymph nodes); RgS, retroglandular submandibular (lymph nodes); IcS, intracapsular submandibular (lymph nodes); SubMe, submental (lymph nodes); AC, anterior cervical (lymph nodes); PC, posterior cervical (lymph nodes); R, right; L, left.

Lymph nodes ranged from 0.4 cm to 2.7 cm in diameter, with no statistically significant differences between genders. There were 34 lymph nodes measuring approximately 1 cm at their greatest diameter. The largest lymph node analyzed was an extraglandular parotid lymph node, which measured 2.7 cm.

We observed anterior cervical lymph nodes in all 20 subjects, identifying a total of 29 such lymph nodes. Of those 29 lymph nodes, 21 (72.5%) had a diameter ≥ 1 cm. Similarly, posterior cervical and intraglandular parotid lymph nodes were observed in 19 (95%) of the subjects.

Among the females, the superficial parotid and left extraglandular parotid lymph nodes had the smallest mean diameters. Those with the largest mean diameters were the right retroglandular submandibular lymph nodes and the left anterior cervical lymph nodes (1.5 cm and 1.3 cm, respectively). The lymph nodes with the largest standard deviations of their mean diameters were the right extraglandular parotid lymph nodes and the right retroglandular submandibular lymph nodes (1.7 cm and 1.5 cm, respectively). The smallest standard deviations were observed for the left superficial parotid lymph node and the left extraglandular parotid lymph node (0 cm for both).

Among the males, the lymph nodes with the highest mean diameter (1.35 cm) were the right retroglandular submandibular lymph nodes, whereas those with the lowest mean diameter (0.1 cm) were the superficial parotid lymph nodes. The standard deviation of the mean diameter was largest (0.9 cm) for the right extraglandular parotid lymph node, whereas it was lowest (0 cm) for the superficial parotid lymph node.

## DISCUSSION

There are two main findings of this study. First, ultrasound showed that the lymph nodes of the head and neck were elongated, had echogenic hila, and exhibited no abnormalities. Second, lymph nodes were observed distributed in various regions of the head and neck showing great variation in terms of size, ranging from 0.4 cm to 2.7 cm in diameter.

The results of the study show that ultrasound can provide data on the location, size, and echogenicity of lymph nodes of the face and neck in male and female individuals. The subjects who participated in this study did not report any health complaints. Features that can indicate malignancy, such as necrosis, calcification, extracapsular spread, and vascularization^(3)^, were not observed in any of the lymph nodes evaluated. The elongated shape and echogenic hila observed for the cervical lymph nodes evaluated in the present study correspond to what has been described in the literature^(3,5)^.

The method used in the present study, ultrasound, is a useful imaging method for the evaluation of lymphadenopathy and can detect isolated enlarged lymph nodes in lymphocyte chains^(7,8)^. However, the diagnosis of lymphadenopathy is difficult to make without knowledge of the standard of normality^(9,10)^. The present study aimed to add to that knowledge as it applies to the lymph nodes of the head and neck. Some authors have published lymph node classification systems to facilitate the evaluation of cervical lymphadenopathy^(11)^.

In the present study, the mastoid, parotid (extraglandular and intraglandular), submandibular (preglandular, retroglandular, and intracapsular), submental, and cervical (anterior and posterior) lymph nodes were easily observed on ultrasound. The superficial parotid lymph node was observed in only one of the subjects. As has previously been reported^(4)^, certain lymph nodes could not be observed on ultrasound, examples including the facial, sublingual, and retropharyngeal lymph nodes.

In the present study, the diameter of the lymph nodes ranged from 0.4 cm (in 0.9% of the subjects) to 2.7 cm (an extraglandular parotid lymph node in an 18-year-old patient), with no statistically significant differences between genders. Most lymph nodes (15.8%) measured approximately 1 cm in diameter. The lymph nodes most often identified were the anterior cervical lymph nodes (in 72.5% of the subjects), as well as the posterior cervical and intraglandular parotid lymph nodes (in 95% of the patients).

Among the female subjects, the mean diameter was smallest for the parotid lymph nodes, whereas it was largest for the right retroglandular submandibular and left anterior cervical lymph nodes. The right retroglandular submandibular lymph nodes also had the largest mean diameter in the male subjects, among whom the superficial parotid lymph nodes had the smallest mean diameter.

Because of their location and size, the lymph nodes in the following groups were not detectable on ultrasound: the infraorbital/zygomatic group (lateral/medial retropharyngeal and deep lateral cervical lymph nodes); the prelaryngeal group; the prethyroid group; the pretracheal/paratracheal group (prevascular/retrovascular submandibular, lateral/ medial sublingual, and facial); the mandibular group; and the buccinator group. That is in accordance with the literature consulted^(4)^.

In conclusion, the lymph nodes on both sides of the face and neck can be examined by ultrasound in men and women. Using that imaging method, we were able to observe lymph nodes with diameters smaller than 0.4 cm.
